# Deep learning for discriminating non-trivial conformational changes in molecular dynamics simulations of SARS-CoV-2 spike-ACE2

**DOI:** 10.1038/s41598-024-72842-w

**Published:** 2024-09-30

**Authors:** Lucas Moraes dos Santos, José Gutembergue de Mendonça, Yan Jerônimo Gomes Lobo, Leonardo Henrique Franca de Lima, Gerd Bruno Rocha, Raquel C. de Melo-Minardi

**Affiliations:** 1 Department of Computer Science, Federal University of Minas Gerais, Belo Horizonte, Minas Gerais Brazil; 2 Department of Chemistry, Federal University of Paraíba, João Pessoa, Paraíba Brazil; 3 Department of Exact and Biological Sciences, Federal University of São João Del Rei, São João del Rei, Minas Gerais Brazil

**Keywords:** Molecular dynamics, Distance maps, Deep learning, CNNs, Machine learning, Molecular dynamics

## Abstract

Molecular dynamics (MD) simulations produce a substantial volume of high-dimensional data, and traditional methods for analyzing these data pose significant computational demands. Advances in MD simulation analysis combined with deep learning-based approaches have led to the understanding of specific structural changes observed in MD trajectories, including those induced by mutations. In this study, we model the trajectories resulting from MD simulations of the SARS-CoV-2 spike protein-ACE2, specifically the receptor-binding domain (RBD), as interresidue distance maps, and use deep convolutional neural networks to predict the functional impact of point mutations, related to the virus’s infectivity and immunogenicity. Our model was successful in predicting mutant types that increase the affinity of the S protein for human receptors and reduce its immunogenicity, both based on MD trajectories (precision = 0.718; recall = 0.800; $\hbox {F}_1$ = 0.757; MCC = 0.488; AUC = 0.800) and their centroids. In an additional analysis, we also obtained a strong positive Pearson’s correlation coefficient equal to 0.776, indicating a significant relationship between the average sigmoid probability for the MD trajectories and binding free energy (BFE) changes. Furthermore, we obtained a coefficient of determination of 0.602. Our 2D-RMSD analysis also corroborated predictions for more infectious and immune-evading mutants and revealed fluctuating regions within the receptor-binding motif (RBM), especially in the $\beta _{1}^{\prime }/\beta _{2}^{\prime }-C$ loop. This region presented a significant standard deviation for mutations that enable SARS-CoV-2 to evade the immune response, with RMSD values of 5Å in the simulation. This methodology offers an efficient alternative to identify potential strains of SARS-CoV-2, which may be potentially linked to more infectious and immune-evading mutations. Using clustering and deep learning techniques, our approach leverages information from the ensemble of MD trajectories to recognize a broad spectrum of multiple conformational patterns characteristic of mutant types. This represents a strategic advantage in identifying emerging variants, bypassing the need for long MD simulations. Furthermore, the present work tends to contribute substantially to the field of computational biology and virology, particularly to accelerate the design and optimization of new therapeutic agents and vaccines, offering a proactive stance against the constantly evolving threat of COVID-19 and potential future pandemics.

## Introduction

In bioinformatics, emerging solutions have transformed the growing volume of biological omics data into knowledge, through methodologies based on machine learning^1^. Deep learning (DL), a subfield of machine learning, has been successful in extracting meaningful patterns from data, as it can handle high-dimensional representations of data by modeling high-level abstractions^2^. This is achieved from multiple nonlinear transformations in neural networks, where the output of one node is connected to the input of each succeeding node. This arrangement of neuronal units forms dense layers, constituting a fully connected network^3^.

In general, deep learning-based pipelines for molecular dynamics simulations are composed of a feature engineering step that reduces the high-dimensional structure space to a low-dimensional feature space; pre-processing that adapts the simulation data to a representation appropriate for algorithm input; a deep neural network composed of feature extraction and classification modules; and a stage of evaluation and interpretability of the predictions made by the model^2^.

In structural bioinformatics, convolutional neural networks (CNNs) have been widely used to predict protein structure or function^1^, which can be attributed to two primary aspects: (i) CNNs benefit from the symmetries of structural representations (e.g., distance maps) through translation invariance, effectively learning patterns regardless of their spatial location in the input; (ii) convolutional layers primarily capture local dependencies within their receptive fields through learned filters, focusing on small spatial regions of the input rather than the entire global structure at once^2,4,5^. These architectures have represented the state-of-the-art in structure prediction^1,6^ and in the identification of functional states from MD trajectories^7–9^.

Molecular dynamics (MD) is another relevant computational method to investigate the dynamic behavior of biomolecular systems at the atomic level^10^. MD simulations combined with experimental data have allowed the investigation of biological processes that would be difficult or even impossible to observe experimentally^11^.

Given the voluminous and high-dimensional nature of MD trajectories, advances in MD simulation analysis applied to molecules, such as cluster analysis, have enabled more efficient exploration of the conformational states in protein complexes^12^. Integrating this method with DL-based approaches improves the prediction of intrinsic protein movements, reducing the risk of overlooking subtle but significant conformations, in which manual analysis could result^9^.

Moreover, remarkable progress has been made in the use of methodologies for in-depth analysis of MD trajectories. For example,^7^ and^9^ combined pixel representations and CNNs to identify functional states in MD trajectories of G protein-coupled receptors (GPCRs), as well as the residues underlying the active states, elucidating the activation mechanisms of these receptors. Both studies transform data extracted from trajectories, including atom coordinates (*x*, *y*, and *z*), into pixel-based representations using RGB components, generating a representation known as pixel maps. Although the model was successful at classifying the ligands, the pixel representation has the disadvantage of being sensitive to translational and rotational movements of proteins, requiring additional preprocessing of the trajectory to remove bias.

Distance maps (DMs) have provided an alternative to this problem as they are invariant to protein rotation and translation. By using distance maps to reduce high-dimensional MD data while preserving crucial spatial information, we gain an advanced approach for analyzing the dynamic behavior of proteins over time. The inherent equivariance of distance maps enables the model to generalize more effectively to unseen structures, regardless of their orientation or position, compared to the purely geometric^13^ or pixel-based representations^7,9^ used in earlier studies. This approach also minimizes the need for extensive preprocessing to remove bias or additional data augmentation steps to ensure robustness against transformations. Furthermore, their low dimensionality is desirable in AI applications^5^.

Recently, 3D ResNets have been used to identify conformational changes associated with spatiotemporal patterns of ligands bound to the $\beta$2-adrenergic receptor ($\beta$2AR), a specific type of GPCR^8^. For this purpose, the model used time series of protein distance maps (PDMs) derived from the trajectories of $\beta$2AR-ligand complexes, as input. The study revealed that ligands of the same type tend to share the same dynamics, characterized by conformational changes throughout the GPCR system induced by residues at the binding site^8^.

Despite the relevant contributions of these works, it is possible to observe that the analyzed conformations involve significant rearrangements within the protein structure, often referred to as large-scale conformational changes^8,9^. However, small localized changes, such as loop movements within protein motifs, are crucial for protein stability, functionality, and ligand binding and merit significant attention.

In this study, we focus on the Spike (S) protein of SARS-CoV-2 (severe acute respiratory syndrome coronavirus 2) as a case study and model the trajectories from MD simulations of the SARS-CoV-2 S protein interacting with human receptor, specifically targeting the receptor binding domain (RBD) and represent them as interresidue distance maps. Subsequently, we developed a deep learning-based system to predict the functional impact of point mutations that could potentially increase the protein’s affinity for the human receptor while reducing its immunogenicity.

Our hypothesis is that specific point mutations in the RBD region should promote non-trivial conformational changes that are indicative of certain variants or even entire lineages of the virus. To support this hypothesis, a literature review was performed. The results highlight the importance of these mutations in the conformation of the virus and its pathogenicity^14–17^. In vitro data show that mutations in the SARS-CoV-2 and SARS-CoV-1 RBDs are capable of evading neutralizing antibodies^18,19^. In addition to structural changes, we discuss how the proposed conformational changes can influence transmissibility and immune response, highlighting the importance of these mechanisms for effective control of viral spread.

Some SARS-CoV-2 strains can increase infectivity and transmissibility, posing a challenge for antiviral drug and vaccine projects against COVID-19 in a short period of time. Because it is a hypermutable virus, its transmissibility tends to favor the emergence of variants with critical mutations and shared zoonotic biological characteristics. Therefore, identifying the patterns that characterize the conformations exhibited by these variants could help to accurately predict potential strains linked to the virus. These findings could be valuable in the development of new drugs and vaccines against COVID-19 or even for future epidemics.

Previous research has successfully employed graph neural networks^13^ or self-attention mechanisms^20^ to predict changes in binding affinity between RBD and ACE2, without requiring MD simulations beyond training data. However, these architectures require structural information from both viral and human proteins, making them dependent on the complex. Furthermore, the effects of some mutations (e.g., S:V367F, S:S477N) may be time-dependent and emerge only after prolonged molecular interactions, requiring simulations of the order of nanoseconds or longer to fully capture their functional impact^21–24^.

The inherent flexibility of the SARS-CoV-2 spike protein (S) is pivotal in modulating its binding affinity to the ACE2 receptor, directly influencing viral virulence^22^. Several studies underscore the importance of this dynamic adaptability, particularly within the receptor-binding motif (RBM), for optimizing interaction with the human receptor^25^. Molecular dynamics (MD) simulations have revealed a heightened flexibility in the receptor binding domain (RBD) of SARS-CoV-2 compared to its counterpart in SARS-CoV, notably in key regions of ACE2 recognition comprising residues Gln474—Gly485, Cys488—Phe490 and Ser494— Tyr505^24,26^. These simulations provide a nuanced understanding of the dynamic ACE2-RBD interaction network, revealing subtle conformational changes (e.g, flexible loops) that remain elusive to static structural analyzes^22,27,28^.

In this sense, the integration of MD simulations offers a comprehensive understanding of the dynamic interaction landscape between the SARS-CoV-2 spike protein’s receptor-binding domain (RBD) and the ACE2 receptor^29^. This approach reveals a broader spectrum of conformational patterns across various mutant types, making it crucial for unraveling the time-dependent structural evolution of RBD. Such insights are crucial to understanding the mechanisms of viral adaptation and immune evasion in the context of emerging variants^30^.

Furthermore, we verified whether it is possible to anticipate the effects of mutations by analyzing the most probable conformation along the MD trajectory. This approach avoids the need for a large number of simulation frames as input to the AI model, even though the model has been trained with MD trajectories.

## Methods

### System preparation and molecular dynamics protocol

We obtained the crystal structure of the SARS-CoV-2 spike receptor binding domain (RBD) bound to angiotensin-converting enzyme 2 (ACE2) from PDB: 6M0J and selected amino acid residues 333 to 527, corresponding to the RBD, using PyMOL (PyMOL Molecular Graphics System, Version 2.4 Schrödinger LLC)^31^. In total, we performed 37 systems based on the RBD FASTA sequence and used a homology model to determine the 3D structures of proteins in SWISS-MODEL^32^.

We estimated protonation states for all residues in both systems from a standard physiological blood pH of 7.4, a salinity of 0.15 M, and internal and external relative permittivities of 10 and 80, respectively, using the H${++}$ web server^33^. We parameterized the systems using the tleap tool from AmberTools21^34^ and solvated them in a cubic box with a minimum edge distance of 12Å. We describe the water, protein, and glycidic fractions of the systems with the AMBER force fields TIP3P, ff14SB, and GLYCAM-06, respectively.

We also added ions $Na^{+}$ and $Cl^{-}$ to reach a physiological salinity of 0.15 M. After solvation, minimization, equilibration, and productive MD simulation steps were performed using NAMD 2.13 CUDA verbs^35^. with the AMBER force field. We performed all simulations with a time step of 1 fs using an NPT ensemble, with the temperature and pressure kept constant at 310 K and 1 atm, respectively, by means of a Langevin thermostat and a Langevin piston. We used periodic boundary conditions and a 10 Å electrostatic interaction cut-off point for non-bonded interactions and calculated the long-range electrostatic interactions using Particle Mesh Ewald.

A relaxation protocol was applied under NPT conditions to all systems before performing the productive MD simulation, and a multistep equilibration protocol was used. This protocol consisted of (1) 500 minimization steps with harmonic constraints on protein atoms; (2) 500 steps of unconstrained minimization; (3) 300 ps equilibrium with harmonic constraints on protein atoms; (4) 300 ps equilibrium with harmonic constraints on the backbone atoms; (5) 300 ps of unrestricted balance; and (6) 1 ns preproductive MD simulation with reset speeds and no restrictions. Finally, we performed three independent 100 ns MD simulations for each S RBD protein system with the corresponding mutations on final relaxation coordinates on the SDumont supercomputer at the Brazilian National Laboratory for Scientific Computing (LNCC).

### Trajectory analysis

We performed a root mean square fluctuation analysis per residue (RMSF) against the average frame for the $C\alpha$ carbons, aligning the entire RBD with the least mobile residues (333–437, 454–456, 492–494 and 509–526). We estimated RMSFs for each of the three replicates individually, as well as for their combined data. Additionally, to evaluate the convergence between the different simulations of each system, two-dimensional (2D) root mean square deviation (2D-RMSD) plots relative to the protein backbone were generated using the cpptraj plug-in^36^ of AmberTools 21^34^.

### Database

Our database consists of 38 (thirty-eight) distinct systems, each derived from MD simulations, with most systems containing between 1 (one) and 3 (three) point mutations per mutant (a comprehensive table listing all systems utilized in this study, including their specific RBD mutations, is presented in Supplementary Table 1). Among these, 17 (seventeen) were selected for machine learning-based analyzes, which involved approximately 85K frames; These included the original 2019-nCoV strain, also known as the “wild type” (WT)^37^; neutral mutants; and variants currently monitored by the WHO (VBM)^38^. These variants, previously classified as variants of concern (VOCs) or variants of interest (VOIs), are known for their increased infectivity and transmissibility; We assign the label “$++$” to the corresponding systems (Table 1).

**Table 1:** Database composition.

WHO label	Nextstrain clade	Pango lineage	Current status	Co-mutations in RBD	Label	References
Beta	20H	B.1.351	VBM	K417N, E484K, N501Y	$++$	^39,40^
Gamma	20J	P.1	VBM	K417T, E484K, N501Y	$++$	^41,42^
Delta	21A	B.1.617.2	VBM	L452R, T478K	$++$	^43,44^
Epsilon	21C	B.1.427/9	VBM	L452R	$++$	^45^
Lota	21F	B.1.526	VBM	S477N, E484K	$++$	^46,47^
Kappa	21B	B.1.617.1	VBM	L452R, E484Q	$++$	^48,49^
Mu	21H	B.1.621	VBM	R346K, E484K, N501Y	$++$	^50,51^
Omicron^a^	21K	B.1.1.529	VOC	G339D, S371L, S373P,	$++$	^52^
				S375F, K417N, N440K,		
				G446S, S477N, T478K,		
				E484A, Q493R, G496S,		
				Q498R, N501Y, Y505H		
Theta	21E	P.3	VBM	E484K, N501Y	$++$	^53^

A comprehensive list of variants with WHO Labels^54^, Nextstrain clade^55^, Pango Lineages, current status^38^ and designation in WHO.

^a^ The Omicron variant is the only one with more than 3 (three) point mutations in the RBD^52^

Furthermore, for systems that represent neutral mutants - those that neither increase receptor affinity for ACE2 nor enhance resistance to antibodies - we label them as “${-}{-}$”. The list of single-type mutations, presented in the *S:mutation (label)* format^55,56^, is as follows: S:V445L (${-}{-}$), S:K417Y (${-}{-}$), S:N439R (${-}{-}$), S:Q498I (${-}{-}$), S:S494K (${-}{-}$), S:Y489F (${-}{-}$), and S:Y505F (${-}{-}$)^57,58^. We labeled the systems according to the published literature. The wild-type strain was labeled “${-}{-}$”.

Complementarily, we conducted a comprehensive analysis of all molecular dynamics simulations to characterize the systems based on their mutant types, with particular attention to mobility changes in key regions such as the RBD, RBM, and loop regions. In this context, systems with mutant types that exhibit decreased affinity for ACE2 and increased antibody resistance, labeled as “$-+$”, as well as those in which the mutation demonstrates increased affinity for ACE2 and decreased antibody resistance, labeled as “$+-$”, were also analyzed. Table 2 provides a summary of the mutant types present in the database, along with their associated effects on viral binding affinity and immune evasion.

**Table 2:** Impact of mutations on viral binding affinity and immune evasion.

Label	ACE2 affinity	Antibody resistance
$++$	Increased	Increased
${-}{-}$	No Effect	No Effect
$-+$	Decreased	Increased
$+-$	Increased	Decreased

The systems correspond to triplicates of 25,000-frame trajectories (*dcd* files) obtained through 100 ns simulations, totaling 75,000 frames (300 ns). We sampled 5000 frames using a skip of 15 frames. Next, we separated each of these 5000 frames into *.pdb* files and generated 5 (five) clusters from the hieragglo average linkage algorithm^59^, that is, using the average distance between members of two clusters calculated in cpptraj^36^ and a cutoff of 2 Å for each system. Thus, although there is a reduction in the sample space, the most likely conformations tend to occur with greater probability.

### Feature transformation

**Fig. 1:**
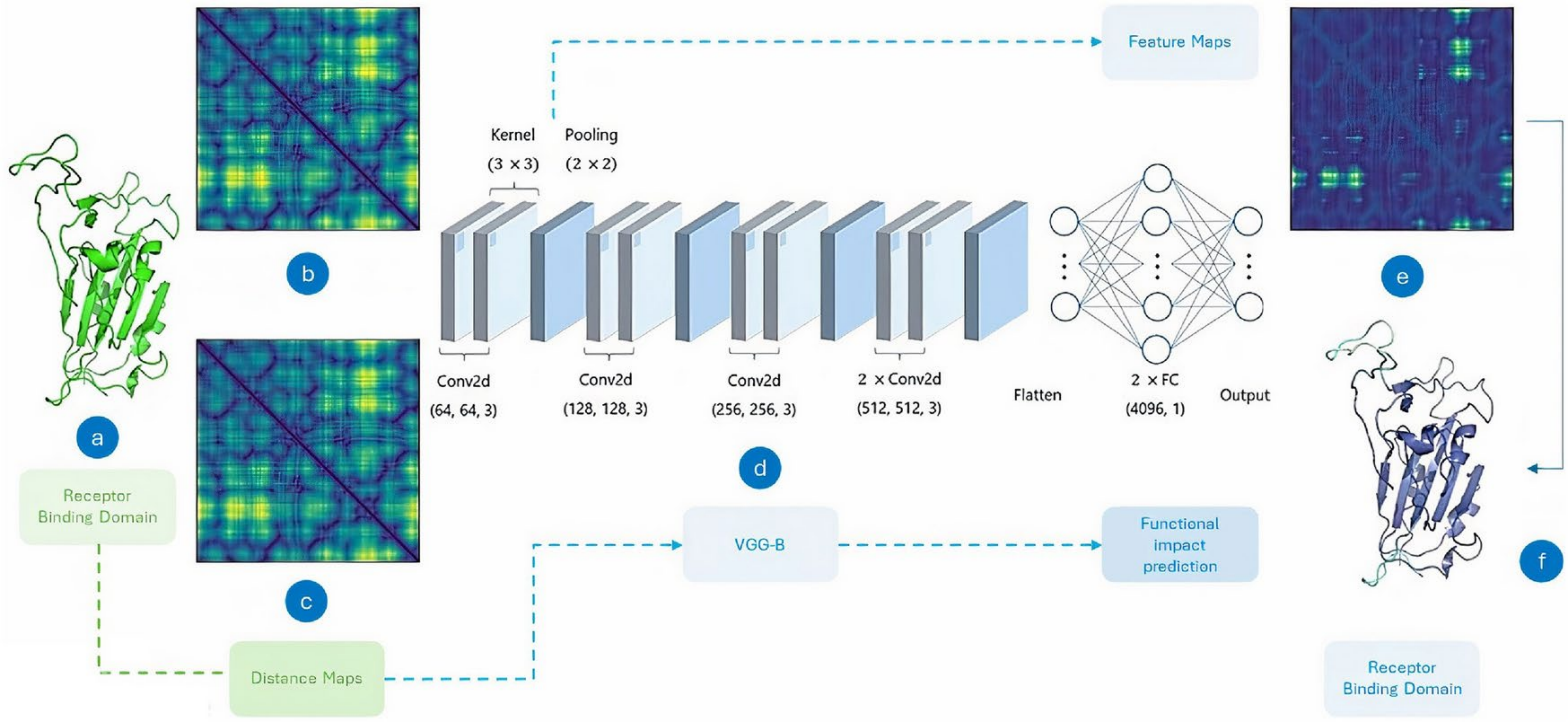
Deep learning-based pipeline. (**a**) Receptor Binding Domain. (**b**) Distance map referring to a frame of the WT MD trajectory. (**c**) Distance map referring to a frame of the Beta variant MD trajectory. It is evident that both maps are indistinguishable to the naked eye, highlighting the intricate nature of the problem. (**d**) VGG-B architecture^64^. (**e**) Feature map extracted from the initial block of conv layers. (Conv1_2), for the centroid of cluster 0 of the Gamma variant. (**f**) Projection of the high-intensity pixels of the feature map onto the 3D structure of the RBD.

Our approach focuses on a restricted data representation to structural information, specifically pairwise distances within the receptor binding domain (RBD) of the SARS-CoV-2 S protein (Fig. 1a). From the MD trajectories, we generate 2D distance maps that serve as the main geometric descriptors of the structure throughout the simulation. Distance maps are graphical representations that detail the spatial arrangements between all pairs of amino acid residues within a molecule, provided as a 2D matrix of the real-valued distances between residues (distance matrices)^60–62^. Consider a set of $N$ points in a three-dimensional space, where each point is defined by its coordinates $(x_i, y_i, z_i)$ and is represented by the position vector $\vec {r}_i$. The Euclidean distance $\delta _{ij}$ between the $i$-th and $j$-th points is defined by the following equation:

1 $$\begin{aligned} \delta _{ij} = {\left\{ \begin{array}{ll} 0, & \text {if } i = j \\ \Vert \vec {r}_j - \vec {r}_i\Vert , & \text {otherwise}, \end{array}\right. } \end{aligned}$$

In the context of structural biology, $\delta _{ij}$ denotes the Euclidean distance between the $i$-th and $j$-th amino acid residue. Therefore, the pairwise distance matrix $[\delta _{ij}]_{N \times N}$ is constructed, where each element $d_{ij}$ represents the Euclidean interresidue distances, $d_{ij}$. The matrix is symmetric, with $\delta _{ij} = \delta _{ji}$ for all $i, j$, and its diagonal elements are zero, indicating that the distance from any residue to itself is zero^60,63^. The complete distance matrix can be represented as

2 $$\begin{aligned} [\delta _{ij}]_{N \times N} = \begin{bmatrix} 0 & \delta _{12} & \delta _{13} & \dots & \delta _{1N} \\ \delta _{21} & 0 & \delta _{23} & \dots & \delta _{2N} \\ \delta _{31} & \delta _{32} & 0 & \dots & \delta _{3N} \\ \vdots & \vdots & \vdots & \ddots & \vdots \\ \delta _{N1} & \delta _{N2} & \delta _{N3} & \dots & 0 \\ \end{bmatrix} \end{aligned}$$

We selected the coordinates (*x, y, z*) of 194 $\alpha$ carbon atoms^5,60^ that constitute the RBD, resulting in distance matrices with dimensions of $194\times 194$ (Fig. 1b,c). The asymptotic computational complexity of this method can be expressed as $O(n^2)$, where *n* indicates the total number of residues in the protein. We converted these matrices into a 2D image (*.png* format) using the Matplotlib library in Python. To ensure compatibility between map dimensions and model input, we resized the dimensions of the maps to $224\times 224$ pixels^64^ by adding padding zeros. We implemented the algorithms for generating the DMs in Python (version 3.9.13).

### CNN-based model development

Since the problem of identifying subtle conformational changes in MD trajectories falls under the *AI-complete* category, and considering that DMs have a grid-like topology^2^, we use the DL technique known as *convolutional neural networks* (CNN)^65^. We choose the VGG architecture, known as Visual Geometry Group^64^ in our methodology. Specifically, we implemented the VGGNet-B variant, characterized by a configuration of 13 trainable layers that include weights.

We represent DMs as 4D tensors, structured based on image dimensions, number of image channels (RGB), and batch size (BS)^66^. In this context, we employ a batch size of 64 samples, as previous studies have shown that batch sizes ranging from 32 to 256 yield improved results^67^, as also observed in related applications^9^. Moreover, we preprocess DM by normalizing the pixel values to a range of $[0-1]$, which is more suitable for efficient processing by neural networks^2^.

Our implementation follows the VGG configuration^64^ (Fig. 1d), except for a slight variation, in which we use a single neuron in the output with a sigmoid activation function, thus transforming the CNN into a binary classifier, resulting in 129 M parameters. After each conv. and pooling layer^64^, we incorporated batch normalization (BN) and rectified linear unit (ReLU) activation. To avoid the problem of internal covariate shift and regularize the model, we added BN before the activation layer^68^.

Additionally, we use dropout after FC layers to mitigate potential overfitting and improve the generalizability of the model^2^. We choose a dropout rate of 0.5, as values within the range of $\left[ 0.3-0.6\right]$ tend to reduce the error rate during training^69^. Dropout is particularly beneficial for datasets exceeding 5K samples, such as the data set of the present problem^69^. We developed the model using well-established ML and neural network libraries such as TensorFlow (version 2.15.0)^70^ and Keras^66^.

### Model training

We partitioned the database into four main subsets of trajectories: WT, VOC, VOI, and neutral. For training the model, we used DMs obtained from MD trajectories related to wild-type (WT) and Beta and Delta variants of concern (VOCs). We selected these variants because they share most of the mutations observed in VOI and are commonly associated with an increased affinity for ACE2 and resistance to natural antibodies or vaccines^71^.

During training, we use cross-validation (CV), a heuristic to minimize the model’s generalization error and optimize hyperparameters^72^. We define a percentage $\gamma < 0.5$ of the training data as a reference to the validation^72^, and used *k*-fold CV.

We randomly partition the training set into *k* mutually exclusive subsets of the same size (*n*/*k*), where *n* is the total number of instances. Thus, validation takes place for one of the subsets, while the remaining $k-1$ serve to train the model. This process occurs *k* successively, and we estimate the average cross-validation error rate (binary cross-entropy loss) as a reference to optimize the model hyperparameters^72^. We set $k = 5$ since $\gamma \ge 0.1$ is commonly recommended and proves effective in various applications^72^. We select the optimal parameter configuration based on the error rate estimates^73^. After adjusting the parameters, we utilize the entire training data set to make predictions on the independent test set.

We conducted the training in Google’s virtual environment, Colab, which provided access to a Jupyter Notebook. The computational resources included an NVIDIA A100 GPU with 40 GB of VRAM and an additional 89.6 GB of RAM.

### Model evaluation

The test set comprises 14 (fourteen) systems, each distributed equally across classes, totaling 70,014 DMs. We evaluated mutations associated with VOIs such as Iota, Kappa, Lambda, Mu, and Theta, as well as VOCs such as Gamma and Omicron. Notably, key mutations including S:L452R, S:T478K, S:E484K, and S:N501Y were commonly observed in most of these systems, leading us to assign them the label “$++$”^41,48,71,74^. Furthermore, we analyzed point mutations S:V445L, S:K417Y, S:N439R, S:Q498I, S:S494K, S:Y489F, and S:Y505F, which are considered neutral^57^, and assigned them the label “${-}{-}$”, thus completing the evaluation of the test set.

During validation, our optimized model achieved an average error rate of 1% with the following specific parameters: learning rate of 1E-3, dropout of 0.5, BS of 64 and 100 training epochs (more details are provided in the Supplementary Material). We used the receiver operating characteristic (ROC) curve (see Fig. 1a in the Supplementary Material) to determine the optimal operating threshold, 0.5, which resulted in a better balance between the recall and false positive rate (1-specificity)^75^.

In the testing, we derived performance metrics from the confusion matrix, including precision (*prec*), true positive rate (TPR)/recall (*rec*), false positive rate (FPR), $\hbox {F}_\beta$ score and Matthews’ correlation coefficient (MCC)^75^. In relation to the $\hbox {F}_\beta$ score, we employ the $\hbox {F}_1$ score, which balances precision and recall by setting $\beta =1$. Furthermore, we calculate the area under the ROC curve (AUC). We calculated complementary metrics such as precision and recall for the test set, since relying solely on the error rate can be limiting as a measure of discriminability. Furthermore, we sought to determine the success rate of the model for each class of problem^75^.

## Results and discussions

### 2D-RMSD analysis of the impact of mutations in the RBD

We developed an analysis that focused on structural variations between the original S protein and its strains, which exhibit different levels of affinity for interaction with the cellular receptor ACE2 and antibodies. In this sense, we generated 2D-RMSD plots to identify regions of high mobility, highlighting differences in conformational sampling between different mutant types in the RBD. Since RBD is the main target of vaccine-induced neutralizing antibodies (nAb), our objective was to understand how these mutations impact interactions between ACE2 and other antibodies^76^. It is relevant to determine whether these mutations compromise the effectiveness of mRNA vaccines and natural immunity, given the ongoing emergence of new variants^40,76^. The following table shows the labels adopted for each mutation phenotype.

**Fig. 2:**
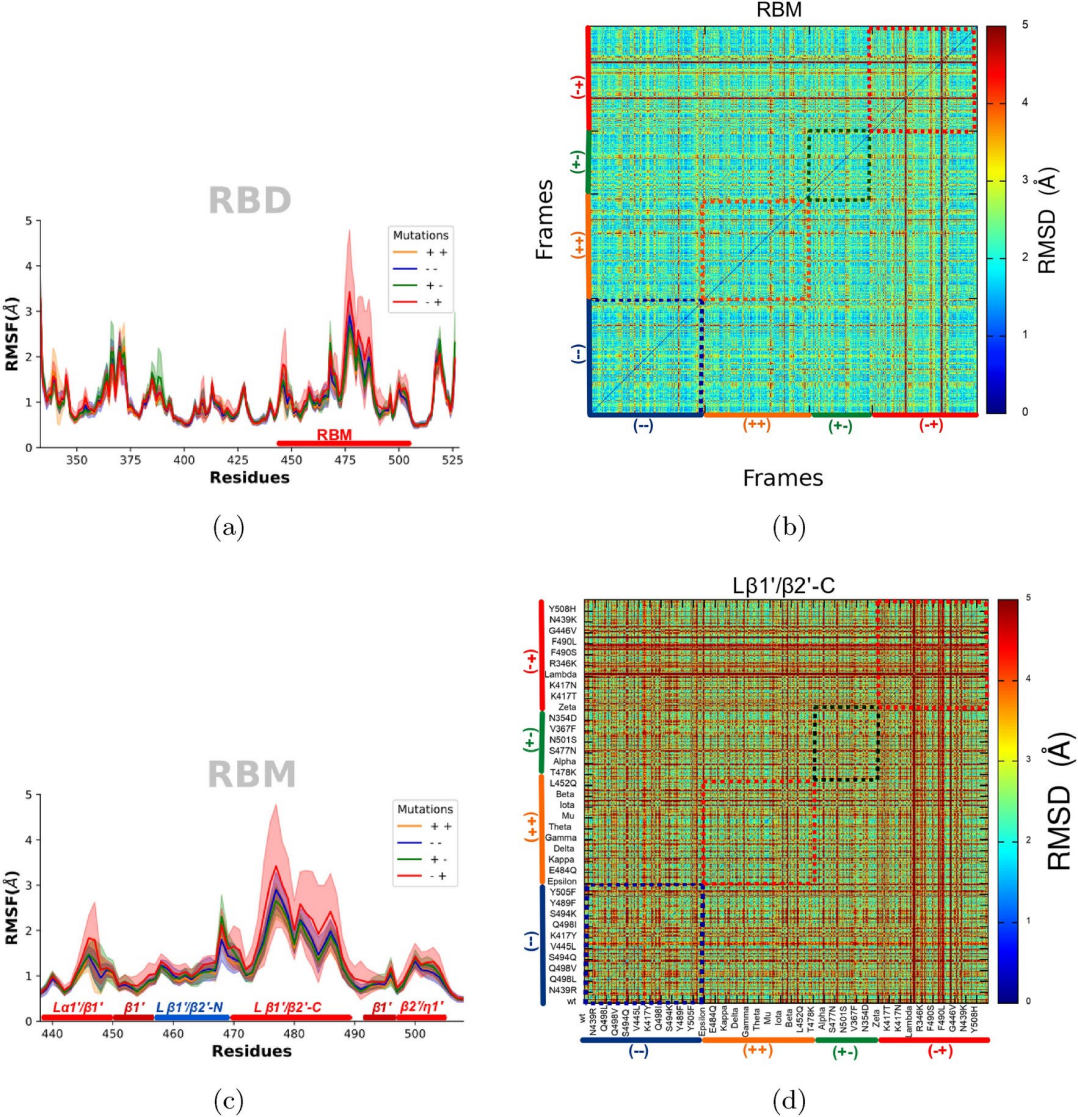
2D-RMSD analysis. (**a**) Root-mean-square fluctuation (RMSF) for the entire receptor binding domain (RBD). (**b**) Two-dimensional root-mean-square deviation (2D-RMSD) graph for the receptor binding motif (RBM). (**c**) RMSF of the RBM region (residues 438–508), aligned and analyzed exclusively within this region. RMSF values are presented as average (lines) and deviation (shading) across three independent replicates, including an aggregated result of all three for each mutation. (**d**) 2D-RMSD graph for the $\beta _{1}^{\prime }/\beta _{2}^{\prime }-C$ loop. The color scheme, ranging from blue to red, represents changes in mobility for the $C\alpha$ atoms of the most mobile ensembles. This coding helps to distinguish the alignment of mutant poses designated as “${-}{-}$”, “$++$”, “$+-$” (mutations more infectious and less resistant to the antibody), and “$-+$”(mutations less infectious and more resistant to the antibody) across all systems and simulations. Each position on the x-axis represents a frame, compared to each frame on the y-axis; the diagonal, always zero, indicates a frame compared against itself.

Our MD analysis of all systems in the database, categorized by their mutant types, revealed that the most significant residue fluctuations and standard deviations were associated with the region spanning residues 360 to 374, as well as along the RBM. especially the $\beta _{1}^{\prime }/\beta _{2}^{\prime }-C$ loop (residues 470 to 490), as illustrated in the RMSF plots (Fig. 2a,c).

In the RBM region, we observed a higher standard deviation for mutations characterized by a lower affinity for interaction with the ACE2 cell receptor and increased resistance to antibodies, labeled “$-+$”. Notably, fluctuations were pronounced in the RBM loops at positions 484 and 501, as indicated in Fig. 2a. These fluctuations were even more marked in the C-terminal extension region of the loop $\beta _{1}^{\prime }/\beta _{2}^{\prime }$, as shown in Fig. 2c. The 2D-RMSD graph presents an all-against-all comparison of molecular dynamics poses, with warmer colors denoting increased mobility. In particular, this loop includes position 484, where the critical mutation S:E484K, common to most VOCs identified by the WHO, occurs. We observed a greater fluctuation in the mobility of the $\beta _{1}^{\prime }/\beta _{2}^{\prime }-C$ loop, particularly associated with the “$-+$” mutations, as shown in Fig. 2b,d. Analyzing these movements relative to the RBD and their internal mobility within the RBM provides significant information.

The magnitude of fluctuation across simulations may be related to mutations that exhibit greater antibody resistance, leading to greater diversity of RBM conformations. This increased structural variability may be a strategy adopted by mutations to ’evade’ the host’s immune response. The same pattern is observed for the key mutation S:N501Y, which exhibits high floating points. In particular, the lambda variant (lineage C.37, mutations S: L452Q and S: F490S) and single-form mutations S: F490S, S: F490L and S: G446V consistently showed RMSD values of 5Å throughout the simulation. These findings suggested that these mutations may help the virus ’escape’ from the immune response and reduce the effectiveness of vaccines, potentially promoting the gain of RBM mobility to evade immune responses^77,78^.

In mutations that increase affinity for the ACE2 interaction and confer antibody resistance, our analyzes revealed a reduced occurrence of conformations compared to the WT strain and neutral mutations denoted “${-}{-}$”. This pattern was clearly visible on the RMSF plots (Fig. 2a,c), particularly within the $\beta _{1}^{\prime }/\beta _{2}^{\prime }-C$ loop (Fig. 2c). Upon analyzing the 2D-RMSD plot, which compares the “$++$” and “${-}{-}$” systems, it is evident that the “${-}{-}$” mutants exhibit a slight increase in mobility within the RBM region, compared to the “$++$” mutations. Notably, the blue square representing the “${-}{-}$” mutations, there is a discernible increase in warmer colors, indicating enhanced mobility close to 5 Å in the RBM. In contrast, the $\beta _{1}^{\prime }/\beta _{2}^{\prime }-C$ loop shows increased mobility for the “${-}{-}$” mutations, compared to the “$++$” mutations, demonstrated by the warmer colors in the 2D-RMSD graph. For the orange square representing the “$++$” mutations, an increase in cooler colors is observed, suggesting reduced mobility near 0 Å. These observations may provide insight into why mutations with increased ACE2-binding affinity in S RBD could influence viral infectivity and immunogenicity. These findings align with those presented in Figs. 2c and 2 of the Supplementary Material.

### Molecular dynamics trajectory classification

As observed in related works^8,9^, we evaluated the generalizability of our predictor to unseen conformations in MD trajectories from an independent test set. These trajectories refer to strains of identical lineage to those included in the training set, in this case belonging to the B.1 lineage^55^. We represent these trajectories using DMs, allowing us to evaluate the predictor’s performance under novel conditions.

To evaluate the potential of a new MD simulation product to match a more infectious and immune-evading variant, it is essential to determine the proportion of correctly predicted instances belonging to the positive class. In this scenario, we use the precision of the positive class, recall, and $\hbox {F}_1$ score to identify trajectories that exhibit distance patterns similar to those observed in the variants present in the training set^75^. These patterns may suggest subtle conformational changes in RBD associated with a gain in affinity for ACE2^15,79–81^, as well as transmissibility^30,82^.

**Fig. 3:**
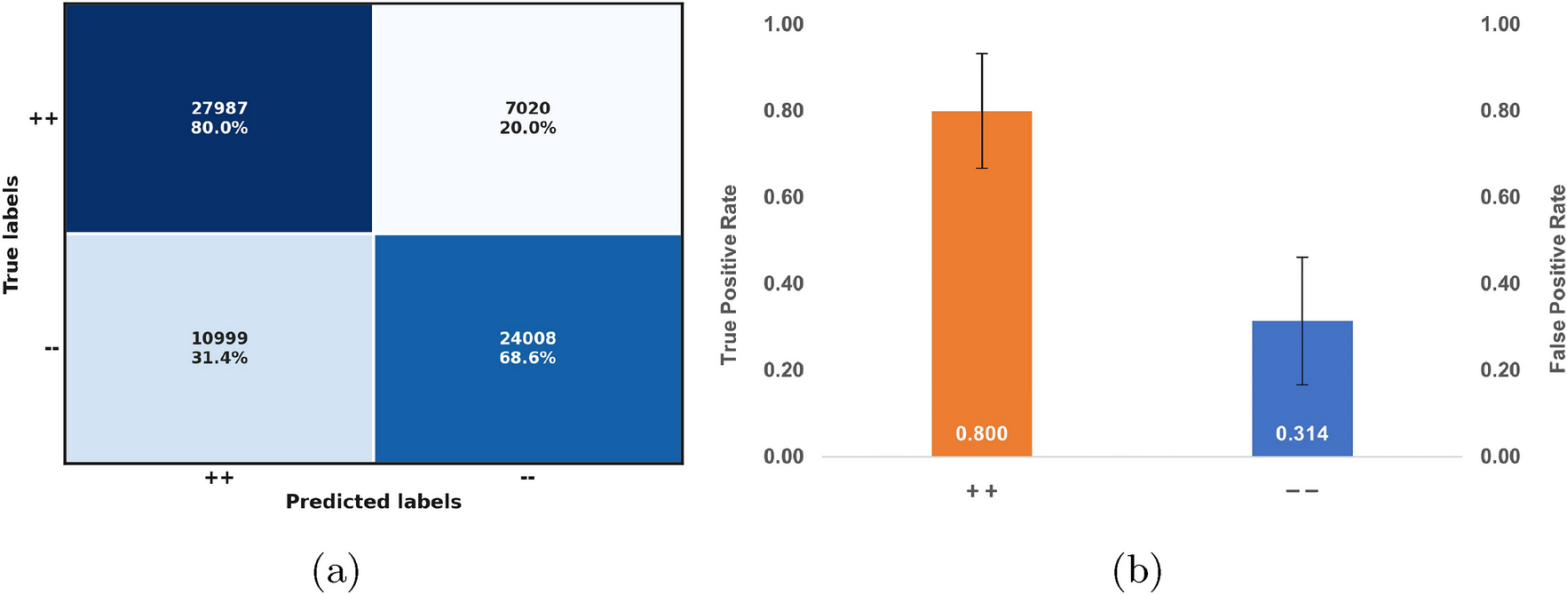
MD trajectories classification. (**a**) Confusion matrix. (**b**) Correctly predicted for the ’++’ class with a corresponding FPR. (Orange) Mean and standard deviation of recall: $0.800 \pm 0.132$; (Blue) Mean and standard deviation of the FPR: $0.314 \pm 0.148$.

Considering the independent test set, we estimate the recall and FPR for the systems assigned the labels $++$ and ${-}{-}$, respectively. Additionally, we calculated the mean ($\mu$) and standard deviation ($\sigma$) of the metrics derived by the model for both classes. Figure 3 summarizes the results, demonstrating the effectiveness of the model in predicting instances with the label $++$. Given $\mu$ and $\sigma$, the recall is considerably higher ($0.800 \pm 0.132$) than the FPR ($0.314 \pm 0.148$), highlighting the need to also reduce the FPR. The model achieved a precision of 0.718, resulting in an $\hbox {F}_1$ score of 0.757, indicating a balanced performance between precision and recall. From the confusion matrix, the MCC was calculated to be 0.488. Furthermore, the estimated AUC was 0.800. These results provide robust evidence of the predictive capacity of the model, particularly for more infectious and less immunogenic mutant types, underscoring its utility to predict the impact of point mutations on the function of the S protein^75^.

### Comparison with state-of-the-art methods

Mutation-induced binding free energy (BFE), represented as $\Delta \Delta G_{\text {Bind}}$ or simply $\Delta \Delta G$, is defined as the difference between the BFE of the mutant type and that of the wild type (WT). Specifically, $\Delta \Delta G_{\text {Bind}} = \Delta G_{\text {Bind}}^{\text {WT}} - \Delta G_{\text {Bind}}^{\text {MT}}$, where $\Delta G_{\text {Bind}}^{\text {WT}}$ is the BFE of the WT and $\Delta G_{\text {Bind}}^{\text {MT}}$ is the BFE of the mutant. This definition reflects the energetic impact of mutation on binding affinity^58,83–85^. Recent studies have established that the transmissibility/infection of viral variants in host cells is proportional to changes in the BFE between the S RBD and ACE2^85^. A positive change in BFE ($\Delta \Delta G_{Bind} > 0$) reveals the ability of the mutation to increase binding between S RBD and ACE2, while a negative change ($\Delta \Delta G_{Bind} < 0$) or a value close to zero ($\Delta G^{MT}_{Bind} \approx \Delta G^{WT}_{Bind}$) suggests reduced or no impact on protein function^57,83,85,86^.

In this context, our objective was to determine the biological significance of our model’s predictions by comparing them with those produced by the TopNetTree model^84,85^, a topology-based network tree methodology specifically developed to forecast changes in the binding free energy of protein-protein interactions (PPI) resulting from mutations. In this study, the authors consolidated more than 1.4 million SARS-CoV-2 genomic sequences from patients and identified 683 point mutations specifically within the receptor binding domain (RBD). They also used a comprehensive library of 130 SARS-CoV-2 antibody structures. Using sophisticated techniques such as viral genotyping, algebraic topology algorithms, and deep learning, they evaluated that RBD comutations influence both binding free energy and antibodies interactions^85^. Their BFE predictions demonstrated a Pearson correlation coefficient of 0.78 when juxtaposed with experimental data^83,86,87^. This high level of precision underscores the potential of these analyses to reliably predict the impacts of viral mutations on vaccine efficacy and antibody neutralization, thereby informing future therapeutic strategies.

**Table 3:** Correlation for model predictions and BFE changes.

WHO label	Co-mutations in RBD^a^	ΔΔ*G* (kcal mon^−1^)	$\Delta \Delta G_{norm}$	${\overline{p}}$
	Y489F	− 0.141	0.253	0.000
	V445L	0.107	0.457	0.250
	K417Y	− 0.450	0.000	0.413
	N439R	− 0.183	0.219	0.444
	Y505F	0.172	0.510	0.458
Gamma	K417T, E484K, N501Y	0.660	0.910	0.603
Epsilon	L452R	0.575	0.840	0.653
Kappa	L452R, E484Q	0.580	0.844	0.712
Mu	R346K, E484K, N501Y	0.768	1.000	0.884
Theta	E484K, N501Y	0.650	0.902	0.950

The values of binding free energy changes, $$\Delta \Delta G$$$$(kcal\cdot mol^{-1})$$, corresponding to neutral mutants and variants, and estimated from the TopNetTree application^84,85^. Additionally, the average probability, $${\overline{p}}$$, resulting from the function of the output layer for the MD trajectories^75^.

^a^ For point mutations, we incorporated data from the ’SARS-CoV-2 Mutation Analyzer’, available online at https://weilab.math.msu.edu/MutationAnalyzer/^86^

**Fig. 4:**
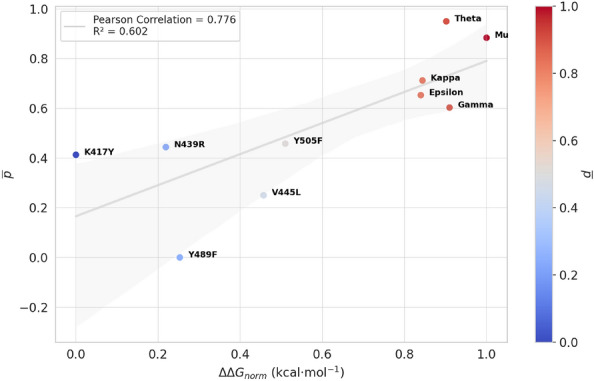
Correlation for model predictions and BFE changes. The estimated product-moment correlation coefficient ($\rho$) indicates a strong positive correlation, with a value of 0.776, and a coefficient of determination ($R^2$) of 0.602. The values of BFE changes ($\Delta \Delta G$ (kcal mol^−1^)) were obtained from TopNetTree application and normalized ($\Delta \Delta G_{norm}$)^84,85^.

We developed our analysis based on changes in BFE (kcal mol^−1^) in the RBD-ACE2 complex, sourced from reputable references^57,85,86,88^. Additionally, for point mutations, we also integrated BFE change data from the online tool “SARS-CoV-2 Mutation Analyzer” (https://weilab.math.msu.edu/MutationAnalyzer/)^86^. Both studies used the TopNetTree model^84^. We calculate the average probability, ${\overline{p}}$, obtained from the output layer’s sigmoid function for the frames of the trajectories, which determines the likelihood of the instances belonging to the positive class. To ensure uniformity and scale the values between 0 and 1, similar to the probability values, we normalize the changes values in BFE using the Min-Max technique, denoted $\Delta \Delta G_{norm}$ (Table 3). Using these calculated values, we developed a scatter plot in which each data point in the scatter plot represents a database-specific mutation or variant, the coordinates representing a tuple of values ${\overline{p}}$ and $\Delta \Delta G_{norm}$ (Fig. 4).

Our analysis identified two distinct clusters: the first contains data points associated with neutral mutations, where changes in BFE exhibit close alignment with changes in WT BFE ($\Delta G^{MT}_{Bind} \approx \Delta G^{WT}_{Bind}$)^57^. The second group included variants correlated with increased viral infectivity and decreased immunogenicity and exhibited significant increases in BFE changes^85^. To quantify the relationship between these groups, we calculated the product-momentum correlation coefficient (also known as Pearson’s correlation coefficient, $\rho$) between the average probability, ${\overline{p}}$ and the normalized change in BFE, $\Delta \Delta G_{norm}$. This produced a strong positive correlation, with $\rho$ at 0.776 and a coefficient of determination ($R^2$) of 0.602 (Fig. 4).

### Prediction from centroids

As mentioned previously, the centroids derived from hierarchical clustering offer the most representative conformations of the structure. Thus, we aimed to estimate the impact of the most infective and least immunogenic types of mutants on the mobility of the RBD on the basis of the most probable conformation represented by the centroid. Considering the centroid during the model prediction, rather than analyzing all frames of the complete MD trajectory, we can reduce the time required for the MD simulation.

Table 4 presents the distribution of frames per cluster, $c_i$, revealing that the first cluster contains the highest number of frames in the MD trajectories. In most systems, the number of frames corresponds to a percentage greater than 60.0%. To determine whether predicting the centroid for the first cluster allows us to predict the class, we calculate the probability (*p*) for each of the *n* centroids. We estimate the average of these probabilities, weighting them by the ratio between the frame frequency of the *i*th cluster and the number of frames in the trajectory (*w*), mathematically described as

3 $$\begin{aligned} {\overline{p}}_w = \frac{\sum \nolimits _{i=1}^n p_i \cdot w_i}{\sum \nolimits _{i=1}^n w_i}. \end{aligned}$$

The results indicate that the prediction of the centroid concerning cluster 0 already corresponds to the mutant type class, a finding supported by the weighted average, ${\overline{p}}_w$. This behavior agrees with the predictions made for the MD trajectories, as the centroid represents the most representative conformation of the cluster.

**Table 4:** Centroid prediction.

System	$c_0$	$c_1$	$c_2$	$c_3$	$c_4$	$p_0$	$p_1$	$p_2$	$p_3$	$p_4$	${\overline{p}}_w$	${\hat{y}}$
Beta	4364	632	2	2	1	1	1	1	0.998	1	1	$++$
Omicron^a^	3312	1043	640	5	1	0.999	1	1	1	1	0.999	$++$
Theta	4947	27	16	10	1	0.999	1	0.999	1	0.999	0.999	$++$
Mu	2765	1157	872	200	7	0.997	1	1	0	0.782	0.958	$++$
Delta	3571	1418	10	1	1	0.999	0.967	0.999	0	0.983	0.990	$++$
Gamma	4601	72	15			0.831	0.989	0.998	0	0	0.782	$++$
S:Q498I	4501	439	51	1	1	0.292	0.995	0.999	0.999	0.999	0.361	$- -$
S:N439R	4006	549	441	4	1	0.077	0.068	0.027	0	0.008	0.072	$- -$
S:K417Y	3026	1860	107	4	4	7.65E−05	0	0.999	0.965	1.02E-04	0.022	$- -$
S:Y489F	3963	675	265	78	22	0.012	0	0	0	0.4	0.011	$- -$
WT	3019	1952	24	5	1	0	0	0	0	0	0	$- -$

For each system in the database it is possible to observe the number of frames per cluster, $c_i$, as well as the predicted probability, $p_i$,for the corresponding centroids. The last column presents the predicted class, ${\hat{y}}$.

^a^ The Omicron variant is the only one with more than 3 (three) point mutations in the RBD^52^

### Feature visualization

We use a technique known as feature visualization to make the learned features explicit by maximizing activation^89^. The aim is to find the input that maximally activates a specific unit. In CNNs, these units correspond to feature maps. From these maps, we can extract the learned patterns from the MD trajectories, allowing for a better understanding of the conformations.

Initially, we considered several approaches developed in related studies, which involved selecting a percentage of frames from the DM trajectory to identify key residues from deep CNNs to correctly predict test frames^7,8^. In our context, clusters provide a simplified analysis of the conformations sampled for mutant types, and each cluster presents a corresponding *centroid* that represents the most representative conformation.

Due to their representativeness, we focused on the centroids of cluster 0 of the MD trajectories used in model training, that is, the WT and Beta variants, in addition to the trajectory referring to the Gamma variant. In the WT trajectory, 60.4% of the frames are in cluster 0, with an average interpoint distance of 1.238 Å ($\pm \, 0.15$ Å). The Beta variant has 87.3% of frames in cluster 0, with an average distance of 1.223 Å ($\pm \, 0.193$ Å). In turn, the Gamma variant has 91.8% of frames in cluster 0, with an average distance of 1.405 Å ($\pm \, 0.319$ Å). We used a 2 Å cutoff distance.

We generated the corresponding DM for the centroids and used it as input to the model. Initially, we extracted an instance of a feature map from the initial conv block. layers (Conv1_2), for the centroid of cluster 0 of the Gamma variant. We observed that the highlighted region in the feature maps corresponds to the C-terminal extension of the loop $\beta _{1}^{\prime }/\beta _{2}^{\prime }-C$ (comprising residues 470–490 of the RBD) (Fig. 1e). In sequence, we projected the high-intensity pixels of the map onto the 3D structure of the RBD (Fig. 1f). We observe that this segment presents greater flexibility than the structure (see Fig. 2 in the Supplementary Material), which is consistent with the results of 2D-RMSD analyses and corroborated by previous studies.

## Conclusions and perspectives

The application of MD simulations to the analysis of conformational states and functional mechanisms of macromolecules is a well-established approach. However, due to their high dimensionality and large scale, analyses involving MD data typically pose substantial computational demand. In this study, we represent the trajectories of MD simulations of the SARS-CoV-2 spike protein-ACE2 as 2D distance maps, and combine cluster analysis and convolutional neural networks for identifying discriminative and nontrivial conformational changes in these trajectories.

Our model was successful in predicting the functional impact related to mutant types that increase the affinity of the S protein for ACE2 and reduce its immunogenicity, considering both MD trajectories ($prec = 0.718$; $rec = 0.800$; $\hbox {F}_1 = 0.757$; MCC = 0.488; AUC = 0.800) and its centroids. We also obtained a strong positive Pearson correlation between the average probability of sigmoid for MD trajectories and the $\Delta \Delta G_{Bind}$ corresponding to neutral mutants and variants, represented by $\rho$ equal to 0.776. Furthermore, we obtained a coefficient of determination ($R^2$) of 0.602.

We also observed a strong positive Pearson correlation ($\rho = 0.776$) between the average probability of the sigmoid function for MD trajectories and the $\Delta \Delta G_{\text {Bind}}$ corresponding to neutral mutants and variants. Furthermore, we achieved a coefficient of determination ($R^2 = 0.602$). Our 2D-RMSD analysis aligned with the visualization of features for more infectious and immune-evading mutant types and revealed fluctuating regions within the RBM, especially in the $\beta _{1}^{\prime }/\beta _{2}^{\prime }-C$ loop. This region presented a significant standard deviation for mutations that allow SARS-CoV-2 to evade the immune response, with RMSD values of 5Å.

The proposed method represents an efficient alternative to identify potential SARS-CoV-2 strains, which may be potentially linked to more infectious and transmissible mutations. Our approach utilizes clustering and deep learning techniques to extract valuable insights from ensembles of MD trajectories, enabling recognition of a wide range of conformational patterns characteristic of mutant types. This provides a significant advantage in the early identification of emerging variants. Although MD simulations continue to supply the detailed structural data required by the model, our deep learning framework significantly minimizes the need for time-consuming post-simulation analyses. Furthermore, when we consider that the most frequently observed conformation in an MD trajectory often resembles the average conformation in X-ray crystallography or even in NMR experiments, this approach could identify variants of a single structure obtained from these methods without the need for a simulation.

Furthermore, our approach provides a novel perspective by demonstrating that the increase in infectivity and immune evasion associated with spike protein mutations is not solely attributable to direct protein-receptor contacts but is also significantly influenced by intrinsic changes in protein mobility. By considering the dynamic flexibility of the protein, our study offers a relevant understanding of the underlying mechanisms driving viral evolution.

The ability to accurately predict the increased binding affinity of the SARS-CoV-2 spike protein’s RBD to the human ACE2 receptor without any structural data from the ACE2 protein being provided to the learning algorithm. This is achieved by focusing solely on changes in spike protein mobility, representing a methodological advancement not explored in previous studies. This capability highlights the model’s effectiveness in predicting functional impacts based solely on dynamic protein behavior, offering a novel perspective on protein interaction analysis.

The present work tends to contribute substantially to the field of computational biology and virology, particularly to accelerate the design and optimization of new therapeutic agents and vaccines, offering a proactive stance against the constantly evolving threat of COVID-19 or future pandemics. Our findings indicate that the deep learning model can serve as a viable alternative to expensive energy calculations, providing a level of accuracy comparable to that of MD simulation analyses.

## Supplementary Information

Supplementary Information.

## Data Availability

The results relating to the analyses conducted as well as the source code are available in the public repository: https://github.com/LBS-UFMG/MD-ML-Project. The data used during this research are available from authors G.B.R (gbr@quimica.ufpb.br) and R.C.M. (raquelcm@dcc.ufmg.br) on request.

## Abbreviations

2019-nCoV: Novel coronavirus
$$\beta$$2AR: $$\beta$$2-Adrenergic receptor
ACE2: Angiotensin-converting enzyme 2
AI: Artificial intelligence
BFE: Binding free energy
BN: Batch normalization
BS: Batch size
CNN: Convolutional neural networks
COVID-19: Coronavirus disease 2019
CV: Cross-validation
DM: Distance maps
DL: Deep learning
FPR: False positive rate
GPCR: G protein-coupled receptors
LNCC: Brazilian National Laboratory for Scientific Computing
MD: Molecular dynamics
ML: Machine learning
mRNA: Messenger ribonucleic acid
nAbs: neutralizing antibodies
NMR-spectroscopy: Nuclear magnetic resonance spectroscopy
NPT: Constant-temperature, constant-pressure
PPI: Protein–protein interactions
RBD: Receptor-binding domain
RBM: Receptor-binding motif
3D-ResNet: 3D residual networks
ReLU: Rectified linear unit
RMSD: Root-mean-square deviation
RMSF: Root-mean-square-fluctuation
ROC: Receiver operating characteristic curve
S protein: Spyke protein
SARS-CoV-2: Severe Acute Respiratory Syndrome Coronavirus 2
VBM: Variants being monitored
VGGNet: Visual Geometry Group Network
VOC: Variants of concern
VOI: Variants of interest
TopNetTree: Topology-based network tree
TPR: True positive rate
WHO: World Health Organization
WT: Wild-type
