# Supplementary material for: Deep learning for discriminating non-trivial conformational changes in molecular dynamics simulations of SARS-CoV-2 spike-ACE2
Source: Sci Rep. 2024 Sep 30;14:22639. doi: 10.1038/s41598-024-72842-w (PMC11443059; doi:10.1038/s41598-024-72842-w)
Supplement: Supplementary file 1 — Supplementary Information. [file 41598_2024_72842_MOESM1_ESM.pdf]

Supplementary Material for "Deep learning for discriminating non-trivial conformational changes in molecular dynamics simulations of SARS-CoV-2 spike-ACE2"

Lucas Moraes dos Santos[1*], José Gutembergue de Mendonça[2], Yan Jerônimo Gomes Lobo[3], Leonardo Henrique Franca de Lima[3], Gerd Bruno Rocha[2], Raquel Cardoso de Melo-Minardi[1*]

[1*]Department of Computer Science, Federal University of Minas Gerais, Belo Horizonte, Minas Gerais, Brazil.

[2]Department of Chemistry, Federal University of Paraíba, João Pessoa, Paraíba, Brazil.

[3]Department of Exact and Biological Sciences, Federal University of São João Del Rei, São João Del Rei, Minas Gerais, Brazil.

*Corresponding author(s). E-mail(s): lucas.santos@dcc.ufmg.br; raquelcm@dcc.ufmg.br;

Contents

Page 2 **2**
Table 1. Database composition 2

Page 3 **3**
Binary Classification Problem 3

Page 4 **4**
Figure 1. Hyperparameter optimization 4

Page 5 **5**
Figure 2. Feature visualization 5

Page 2

Database Composition

Table 1: Database Composition: WHO Labels [36], Nextstrain Clade [37], Pango Lineage, S RBD Mutations and Corresponding Mutant Types.

WHO Label	Nextstrain Clade	Pango Lineage	RBD Co-Mutations	Label	References
		**Wild Type and Neutral Mutations**			
-	-	B	-	−−	[1]
-	-	-	S:K417Y	−−	[2]
-	-	-	S:N439R	−−	[2, 3]
-	-	-	S:Q498I	−−	[2, 3]
-	-	-	S:Q498L	−−	[2]
-	-	-	S:Q498V	−−	[2]
-	-	-	S:S494K	−−	[2]
-	-	-	S:S494Q	−−	[2]
-	-	-	S:V445L	−−	[2, 4]
-	-	-	S:Y489F	−−	[2]
-	-	-	S:Y505F	−−	[2, 5]
		**More Infective and Immune-Evasive Variants**			
-	-	-	S:E484Q	++	[6–8]
-	-	-	S:L452Q	++	[8, 9]
Beta	20H	B.1.351	S:K417N+E484K+N501Y	++	[10, 11]
Delta	21A	B.1.617.2	S:L452R+T478K	++	[12, 13]
Epsilon	21C	B.1.427/9	S:L452R	++	[14–16]
Gamma	20J	P.1	S:K417T+E484K+N501Y	++	[17–19]
Kappa	21B	B.1.617.1	S:L452R+E484Q	++	[20, 21]
Iota	21F	B.1.526	S:S477N+E484K	++	[22–24]
Mu	21H	B.1.621	S:R346K+E484K+N501Y	++	[25, 26]
Omicron[1]	21K	B.1.1.529	S:G339D+S371L+S373P+S375F+ K417N+N440K+G446S+S477N+ T478K+E484A+Q493R+G496S+ Q498R+N501Y+Y505H	++	[27]
Theta	21E	P.3	S:E484K+N501Y	++	[28, 29]
	**More Infective, Less Immune-Evasive − Less Infective, More Immune-Evasive**				
-	-	-	S:N354D	+−	[14, 30]
-	-	-	S:N501S	+−	[31]
-	-	-	S:N501Y	+−	[32, 33]
-	-	-	S:S477N	+−	[7, 8, 23]
-	-	-	S:T478K	+−	[12, 13]
-	-	-	S:V367F	+−	[14, 30, 34]
-	-	-	S:F490L	−+	[14, 30]
-	-	-	S:F490S	−+	[6–8, 30]
-	-	-	S:G446V	−+	[7, 31]
-	-	-	S:K417N	−+	[7–9]
-	-	-	S:K417T	−+	[7–9]
-	-	-	S:N439K	−+	[3, 14, 30]
-	-	-	S:R346K	−+	[9, 30]
-	-	-	S:Y508H	−+	[14, 30]
Lambda	21G	C.37	S:L452Q+F490S	−+	[30, 35]
Zeta	20B	P.2	S:E484K	−+	[7, 14, 30]

[1] Omicron (21K) has more than 3 mutations in the RBD, as described in [38].

Page 3

Binary Classification Problem

We model the problem of identifying subtle conformational changes in MD trajectories, which are associated with more infectious and immune-evading SARS-CoV-2 strains, as a binary classification. Therefore, we formulate the problem as a test of two mutually exclusive hypotheses:

- H_0, null hypothesis, corresponds to the wild-type (WT)
- H_1, alternative hypothesis, not wild-type (i.e., any of the variants labeled as "++")

Consequently, we can obtain the optimal classifier by the likelihood ratio (Λ) between two hypotheses and a threshold [39], represented by

$$\Lambda(y) = \frac{p(y \mid H_1)}{p(y \mid H_0)} \underset{H_0}{\overset{H_1}{\gtrless}} \lambda. \tag{1}$$

Thus, $p(y \mid H_i)$ is the likelihood function for hypothesis H_i (where $i = \{0, 1\}$), which is evaluated for sample y, and λ is the decision threshold. Thus, for a given sample y, we reject the null hypothesis if the likelihood ratio is greater than a threshold λ, which is independent of y [40].

In the context of the variant identification problem, the class labels are derived from the directory structure, referred to as *negative samples* and *positive samples*. We encoded these labels as float32 scalars with values of 0 or 1, respectively [41]. Consequently, after model prediction, for a given instance $\mathbf{x}$ labeled as 1 (positive sample), the *a posteriori* probability that this instance belongs to the non-wild-type class, $P(\text{non-wild-type}| \, \mathbf{x})$, tends to be closer to 1. A high $P(\text{non wild-type}| \, \mathbf{x})$ indicates significant confidence of the model in its prediction that the instance belongs to the non-wild-type class [42]. Similarly, for an instance $\mathbf{x}$ labeled 0 (negative sample), the a posteriori probability that this instance belongs to the wild-type class, $P(\text{wild-type}| \, \mathbf{x})$, tends to be 0.

Two types of errors can occur in classification. Type I errors represent a failure to reject the null hypothesis, despite being false. Errors of this type are called misses; that is, the model decides that the sample corresponds to the wild-type class when the correct sample is not the wild-type class. In the case of type II errors, the null hypothesis is rejected erroneously. Errors of this type are called false alarms; that is, the model decides that the sample corresponds to the non-wild-type class when the correct sample would be the wild-type class [42].

Therefore, if the alternative hypothesis is true and the model classifies the sample as not wild-type, this indicates that the model correctly infers and does not reject H_1. In hypothesis testing theory, this corresponds to a decision that aligns with the correct class, representing a true positive [42].

Page 4

Hyperparameter Optimization

We employ an automated hyperparameter fitting approach for the model using genetic algorithms (GAs). GAs represent an effective technique for optimizing the topology of a neural network and learning parameters [43]. The objective is to adjust the model capacity by finding the set of hyperparameters within a hypothesis space that optimizes an objective function, that is, the validation error [44].

To determine the optimal hyperparameters, we define a search space that comprises a set of key hyperparameters: BS with options of 32, 64, 128 and 256; a dropout rate of $0.25, 0.3, 0.5$ and 0.6; a number of epochs spanning $50, 100, 150$ and 200; and an Adam optimizer [45] configured with a learning rate ranging between $0.1, 1E-2$, and $1E-3$, with constants $\beta_1 = 0.9$, $\beta_2 = 0.999$, and $\epsilon = 1E-7$ [41]. In this scenario, the individual has been encoded as a vector, where each position on the vector contains a potential value for the hyperparameter. We obtain the fitness of each individual by calculating the average error percentage, considering the performance at all folds.

Once the most adept individuals have been identified based on their fitness, we applied uniform crossover and mutation operations using probabilities of 0.5 and 0.25, respectively [46]. The population was updated over 10 generations. Ultimately, the optimal individual obtained featured hyperparameter ensembles of 64, 0.5, 100, and $1E-3$, corresponding to a cross-validation average error rate of 1%.

In addition, we generate receiver operating characteristic (ROC) curves for the best model configuration based on cross-validation for suboptimal models (Fig. 1a – ROC curve for the best model configuration) and for the independent test set (Fig. 1b – ROC curve for the independent test set). The area under the estimated ROC curve (AUC) for both cases is equivalent to 0.930 and 0.800, respectively. An AUC of 0.800 for the independent test set still reflects a good level of model performance, although this decrease in AUC relative to validation highlights the complexity of the problem.

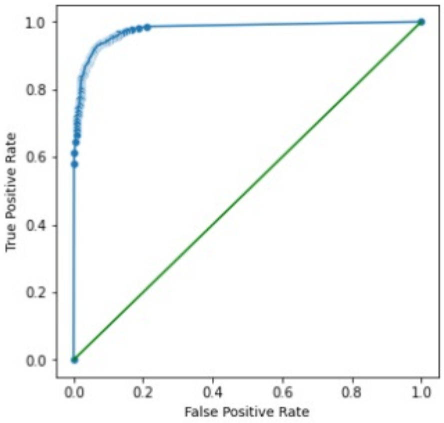

(a)

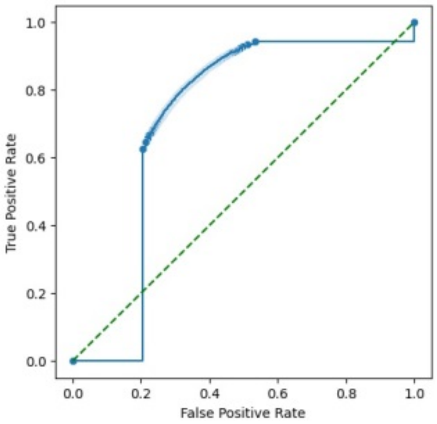

(b)

Fig. 1: Hyperparameter optimization. (a) ROC curve for the best model configuration based on cross-validation. (b) ROC curve for the independent test set.

Page 5

Feature visualization

To better understand the patterns, present in the feature maps, as well as their relationship with fluctuations in the RBD, we generated a 2D visualization to represent pixel intensities relative to feature maps from ImageJ software [47]. For this analysis, we used the centroids that represent cluster 0 of the MD trajectories for both the wild type (−−) and the Beta variant (++). Thus, since each pixel in the feature map corresponds to a specific pair of residues, we can infer that the pixel intensity values are related to pairwise distances.

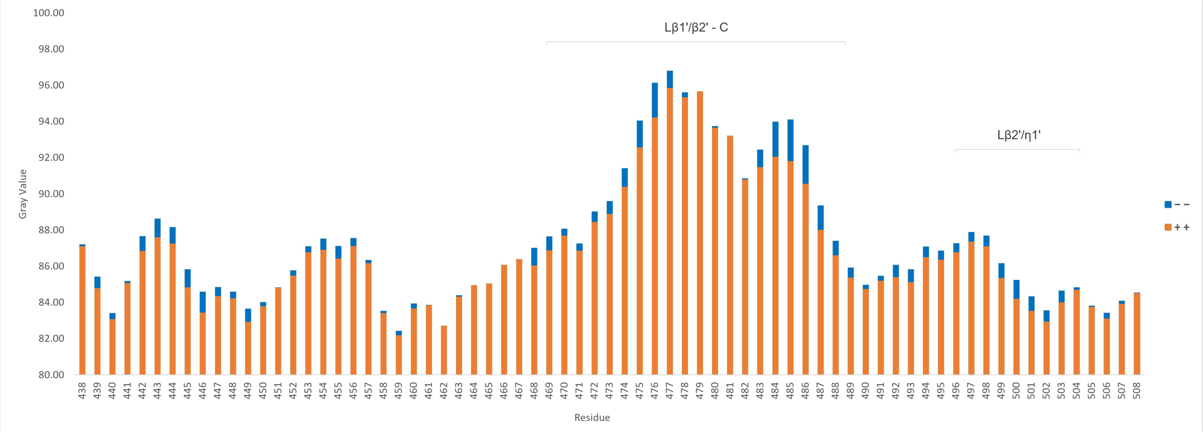

Fig. 2: Plot profile. The superposition of two-dimensional graphs illustrates the averaged pixel intensities of both the WT (−−) and Beta variant (++) centroids' feature maps, with a specific focus on the receptor binding motif (RBM). The region comprising residues 470-490 of the RBD (loop $\beta_1'/\beta_2' - C$) exhibits the most significant difference in mean pixel intensities between the two maps.

In visualization (Figure 2 – Plot profile), the x axis represents the residuals that comprise the RBM region, while the y axis represents the average pixel intensity vertically along the RBM. Upon analyzing the patterns learned by the model, we found that the segment of residuals related to the loop $\beta_1'/\beta_2' - C$ exhibits considerable variations in the intensity of the pixels, when we superimpose the curves on the two centroids. These differences in pixel intensities might be linked to subtle conformational changes resulting from specific mutations, such as S:E484K, which is near the center of the loop region.

References

[1] Wu, F. *et al.* A new coronavirus associated with human respiratory disease in China. *Nature* **579**, 265–269 (2020). 10.1038/s41586-020-2008-3.

[2] López-Cortés, G. I. *et al.* Neutral evolution test of the spike protein of SARS-CoV-2 and its implications in the binding to ACE2. *Scientific Reports* **11** (2021). URL https://doi.org/10.1038/s41598-021-96950-z.

[3] Teng, S. *et al.* Systemic effects of missense mutations on SARS-CoV-2 spike glycoprotein stability and receptor-binding affinity. *Briefings in Bioinformatics* **22**, 1239–1253 (2020). 10.1093/bib/bbaa233.

[4] Alcantara, M. C., Higuchi, Y., Kirita, Y., Matoba, S. & Hoshino, A. Deep mutational scanning to predict escape from bebtelovimab in sars-cov-2 omicron subvariants. *Vaccines* **11**, 711 (2023). URL http://dx.doi.org/10.3390/vaccines11030711.

[5] Chowdhury, R., Boorla, V. S. & Maranas, C. D. Computational biophysical characterization of the sars-cov-2 spike protein binding with the ace2 receptor and implications for infectivity. *Computational and Structural Biotechnology Journal* **18**, 2573–2582 (2020). URL http://dx.doi.org/10.1016/j.csbj.2020.09.019.

[6] Chen, J., Gao, K., Wang, R. & Wei, G.-W. Prediction and mitigation of mutation threats to covid-19 vaccines and antibody therapies. *Chemical Science* **12**, 6929–6948 (2021). URL http://dx.doi.org/10.1039/D1SC01203G.

[7] Harvey, W. T. *et al.* SARS-CoV-2 variants, spike mutations and immune escape. *Nature Reviews Microbiology* **19**, 409–424 (2021). 10.1038/s41579-021-00573-0.

[8] Tao, K. *et al.* The biological and clinical significance of emerging SARS-CoV-2 variants. *Nature Reviews Genetics* **22**, 757–773 (2021). 10.1038/s41576-021-00408-x.

[9] Wang, R. *et al.* Emerging Vaccine-Breakthrough SARS-CoV-2 Variants. *ACS Infectious Diseases* **8**, 546–556 (2022). 10.1021/acsinfecdis.1c00557.

[10] Radvak, P. *et al.* SARS-CoV-2 B.1.1.7 (alpha) and B.1.351 (beta) variants induce pathogenic patterns in K18-hACE2 transgenic mice distinct from early strains. *Nature Communications* **12**, 6559 (2021). 10.1038/s41467-021-26803-w.

[11] Wang, P. *et al.* Antibody resistance of SARS-CoV-2 variants B.1.351 and B.1.1.7. *Nature* **593**, 130–135 (2021). 10.1038/s41586-021-03398-2.

[12] Mlcochova, P. *et al.* SARS-CoV-2 B.1.617.2 Delta variant replication and immune evasion. *Nature* **599**, 114–119 (2021). 10.1038/s41586-021-03944-y.

[13] Pouwels, K. B. *et al.* Effect of Delta variant on viral burden and vaccine effectiveness against new SARS-CoV-2 infections in the UK. *Nature Medicine* **27**, 2127–2135 (2021). 10.1038/s41591-021-01548-7.

[14] Li, Q. *et al.* The impact of mutations in SARS-CoV-2 spike on viral infectivity and antigenicity. *Cell* **182**, 1284–1294 (2020). 10.1016/j.cell.2020.07.012.

[15] Zhang, W. *et al.* Emergence of a novel SARS-CoV-2 variant in southern california. *JAMA* **325**, 1324 (2021). 10.1001/jama.2021.1612.

[16] Deng, X. *et al.* Transmission, infectivity, and neutralization of a spike l452r SARS-CoV-2 variant. *Cell* **184**, 3426–3437.e8 (2021). URL https://doi.org/10.1016/j.cell.2021.04.025.

[17] Faria, N. R. *et al.* Genomics and epidemiology of the P.1 SARS-CoV-2 lineage in Manaus, Brazil. *Science* **372**, 815–821 (2021). 10.1126/science.abh264.

[18] Naveca, F. G. *et al.* COVID-19 in Amazonas, Brazil, was driven by the persistence of endemic lineages and P.1 emergence. *Nature Medicine* **27**, 1230–1238 (2021). 10.1038/s41591-021-01378-7.

[19] Fujino, T. *et al.* Novel SARS-CoV-2 variant in travelers from brazil to japan. *Emerging Infectious Diseases* **27** (2021). URL https://doi.org/10.3201/eid2704.210138.

[20] Wilhelm, A. *et al.* Antibody-mediated neutralization of authentic SARS-CoV-2 b.1.617 variants harboring l452r and t478k/e484q. *Viruses* **13**, 1693 (2021). 10.3390/v13091693.

[21] McCallum, M. *et al.* Molecular basis of immune evasion by the Delta and Kappa SARS-CoV-2 variants. *Science* **374**, 1621–1626 (2021). 10.1126/science.abl8506.

[22] Annavajhala, M. K. *et al.* Emergence and expansion of SARS-CoV-2 b.1.526 after identification in new york. *Nature* **597**, 703–708 (2021). 10.1038/s41586-021-03908-2.

[23] Zhou, H. *et al.* B.1.526 SARS-CoV-2 Variants Identified in New York City are Neutralized by Vaccine-Elicited and Therapeutic Monoclonal Antibodies. *mBio* **12**, e0138621 (2021). 10.1128/mbio.01386-21.

[24] Yang, W. *et al.* Epidemiological characteristics of the b.1.526 SARS-CoV-2 variant. *Science Advances* **8** (2022). URL https://doi.org/10.1126/sciadv.abm0300.

[25] Halfmann, P. J. *et al.* Characterization of the SARS-CoV-2 b.1.621 (mu) variant. *Science Translational Medicine* **14**, eabm4908 (2022). 10.1126/scitranslmed.abm4908.

[26] Laiton-Donato, K. *et al.* Characterization of the emerging B.1.621 variant of interest of SARS-CoV-2. *Infection, Genetics and Evolution* **95**, 105038 (2021). 10.1016/j.meegid.2021.105038.

[27] Dejnirattisai, W. *et al.* Sars-cov-2 omicron-b.1.1.529 leads to widespread escape from neutralizing antibody responses. *Cell* **185**, 467–484.e15 (2022). URL http://dx.doi.org/10.1016/j.cell.2021.12.046.

[28] Bascos, N. A. D., Mirano-Bascos, D. & Saloma, C. P. Structural analysis of spike protein mutations in the sars-cov-2 p.3 variant (2021). URL http://dx.doi.org/10.1101/2021.03.06.434059.

[29] Haw, N. J. *et al.* Epidemiological characteristics of the SARS-CoV-2 Theta variant (P.3) in the Central Visayas region, Philippines, 30 october 2020–16 february 2021. *Western Pacific Surveillance and Response Journal* **13**, 60–62 (2022). 10.5365/wpsar.2022.13.1.883.

[30] Liu, J. *et al.* Characterization of SARS-CoV-2 worldwide transmission based on evolutionary dynamics and specific viral mutations in the spike protein. *Infectious Diseases of Poverty* **10** (2021). 10.1186/s40249-021-00895-4.

[31] Verma, J. & Subbarao, N. Insilico study on the effect of SARS-CoV-2 RBD hotspot mutants' interaction with ACE2 to understand the binding affinity and stability. *Virology* **561**, 107–116 (2021). 10.1016/j.virol.2021.06.009.

[32] Planas, D. *et al.* Sensitivity of infectious SARS-CoV-2 b.1.1.7 and b.1.351 variants to neutralizing antibodies. *Nature Medicine* **27**, 917–924 (2021). URL https://doi.org/10.1038/s41591-021-01318-5.

[33] Galloway, S. E. *et al.* Emergence of SARS-CoV-2 b.1.1.7 lineage — united states, december 29, 2020–january 12, 2021. *MMWR. Morbidity and Mortality Weekly Report* **70**, 95–99 (2021). URL https://doi.org/10.15585/mmwr.mm7003e2.

[34] Ou, J. *et al.* V367f mutation in SARS-CoV-2 spike RBD emerging during the early transmission phase enhances viral infectivity through increased human ACE2 receptor binding affinity. *Journal of Virology* **95** (2021). 10.1128/jvi.00617-21.

[35] Kimura, I. *et al.* SARS-CoV-2 lambda variant exhibits higher infectivity and immune resistance (2021). URL https://doi.org/10.1101/2021.07.28.454085.

[36] WHO. Tracking SARS-CoV-2 variants. https://www.who.int/en/activities/tracking-SARS-CoV-2-variants/ (2020).

[37] Aksamentov, I. *et al.* Nextclade: clade assignment, mutation calling and quality control for viral genomes. *Journal of Open Source Software* **6**, 3773 (2021). 10.21105/joss.03773.

[38] Chen, J. *et al.* Omicron variant (B.1.1.529): infectivity, vaccine breakthrough, and antibody resistance. *Journal of Chemical Information and Modeling* **62**, 412–422 (2022). 10.1021/acs.jcim.1c01451.

[39] Fukunaga, K. *Introduction to Statistical Pattern Recognition* (Academic Press Professional, Inc., San Diego, CA, United States, 1990).

[40] Duda, R. O., Hart, P. E. & Stork, D. G. *Pattern Classification* (Wiley, New York, NY, United States, 2001).

[41] Chollet, F. *et al.* Keras. https://keras.io (2015).

[42] Webb, A. R. & Copsey, K. D. *Statistical Pattern Recognition* (Wiley, New York, NY, United States, 2011).

[43] Mitchell, T. M. *Machine Learning* 2 edn (McGraw-Hill, New York, USA, 1997).

[44] Goodfellow, I. J., Bengio, Y. & Courville, A. *Deep Learning* (MIT Press, Cambridge, MA, United States, 2016).

[45] Kingma, P. & Ba, J. Adam: A method for stochastic optimization. *3rd International Conference for Learning Representations* 1–14 (2015). ArXiv:1412.6980v9.

[46] Hassanat, A. *et al.* Choosing mutation and crossover ratios for genetic algorithms—a review with a new dynamic approach. *Information* **10**, 390 (2019). 10.3390/info10120390.

[47] Schneider, C. A., Rasband, W. S. & Eliceiri, K. W. NIH image to ImageJ: 25 years of image analysis. *Nature Methods* **9**, 671–675 (2012). 10.1038/nmeth.2089.
